# A novel approach to stabilization of bleeding gastroesophageal varices in infants

**DOI:** 10.1002/jpr3.70018

**Published:** 2025-03-24

**Authors:** Sussette G. Szachowicz, Elyse Kerian, Catherine DeGeeter, Riad Rahhal

**Affiliations:** ^1^ Stead Family Department of Pediatrics University of Iowa Iowa City Iowa USA; ^2^ University of Iowa Roy J and Lucille A Carver College of Medicine Iowa City Iowa USA

**Keywords:** gastric varix, GI bleed, portal hypertension, tamponade

## Abstract

Gastroesophageal variceal bleeding is the most serious complication of portal hypertension. The interventions available including sclerotherapy, variceal banding, and balloon tamponade, are limited by patient age. A 4‐month‐old with congenital cytomegalovirus, cholestasis, splenomegaly presented to the emergency room after two episodes of hematemesis. The patient required a transfusion of packed red blood cells for anemia. Upper endoscopy revealed no active bleeding, four grade 3 esophageal varices with red wale signs, and a single gastric varix. Sclerotherapy into high‐risk varices was completed. Forty‐eight hours later, patient developed re‐bleeding. Upper endoscopy revealed bright red blood in the stomach. A large clot at the gastroesophageal junction was attributed to the gastric varix. Given the age of the patient and small size, endoscopic bleeding control interventions were limited. A foley catheter was placed in an orogastric manner for balloon tamponade. The intervention was a temporizing measure to allow for transfer to a liver transplant center.

## 1 INTRODUCTION

Gastroesophageal variceal bleeding is the most serious complication of portal hypertension and is the most common cause of severe acute upper gastrointestinal bleeding in the pediatric population, with a mortality rate of up to 30%.^1^, ^2^, ^3^, ^4^ The evidence‐based intervention strategies available, including sclerotherapy, injection of cyanoacrylate glue, variceal banding, and balloon tamponade, are limited by patient and endoscope size.^2^ Therefore, stabilization of small infants with gastroesophageal variceal bleeding can be challenging and may require novel approaches.

## 2 CASE REPORT

### 2.1 Initial presentation and management

**Figure 1:**
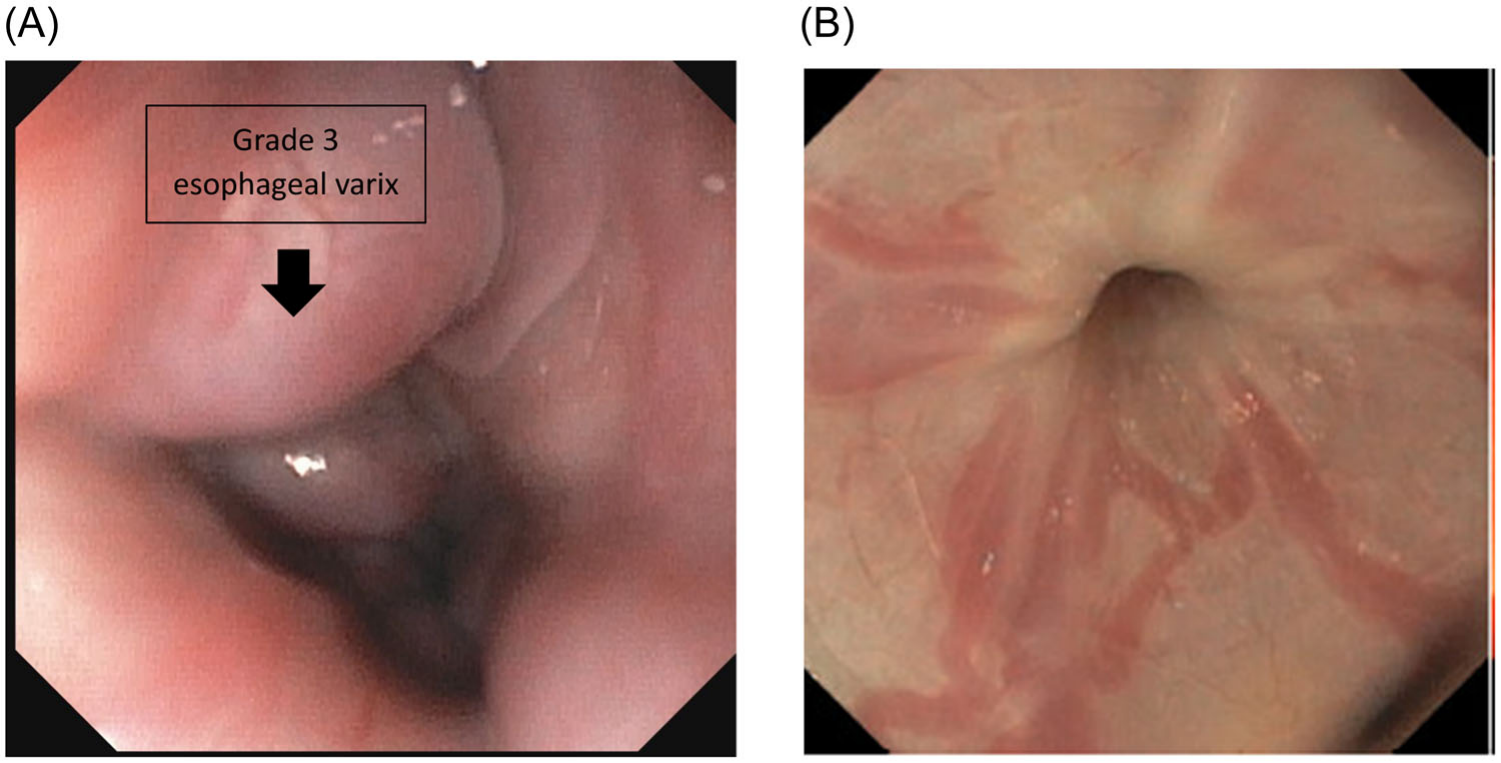
(A) First endoscopy with proximal view of one of the four, grade 3, esophageal varices before intravariceal sclerotherapy performed. (B) Second endoscopy with improved esophageal varices without evidence of active bleed.

A 4‐month‐old male born prematurely at 32 weeks, weighing 4.6 kg, with a history of congenital cytomegalovirus on valganciclovir, cholestasis, intrahepatic calcifications, splenomegaly, adrenal insufficiency, and gastrostomy tube (g‐tube) dependence presented to the emergency room after two episodes of hematemesis with blood coming from his g‐tube. Initial workup was notable for a hemoglobin of 8.9 g/dL (compared to baseline of 9.6–10.9 g/dL) and platelets of 69 k/mm^3^, with decline to 7.6 and 60, respectively, after 6 h. Coagulation studies were within normal limits. A right upper quadrant ultrasound demonstrated splenomegaly without hepatic abnormalities. The patient was admitted to the pediatric intensive care unit (ICU) and shortly thereafter developed frank bloody stools. The infant was made NPO and was given intravenous fluids, pantoprazole 2 mg/kg/day, an octreotide bolus and continuous infusion at 1 mcg/kg/h, ceftriaxone 50 mg/kg, and stress dose steroids. A transfusion of packed red blood cells at 15 cc/kg was then administered, and he was taken to the operating room for urgent upper endoscopy with bleeding control. Endoscopic evaluation showed four large circumferential grade 3 esophageal varices (proximal view of one varix is represented in Figure 1A). In the distal esophagus, red wale signs were noted on 3 of the 4 varices without active bleeding. A single small gastric varix was also observed. Given the patient's age and size, endoscopic interventions for control of variceal bleeding were limited. Thus, intravariceal sclerotherapy with Polidocanol (the available sclerosing agent at that time) was performed into the high‐risk esophageal varices without intraprocedural complications.

### 2.2 Re‐bleeding and secondary intervention

**Figure 2:**
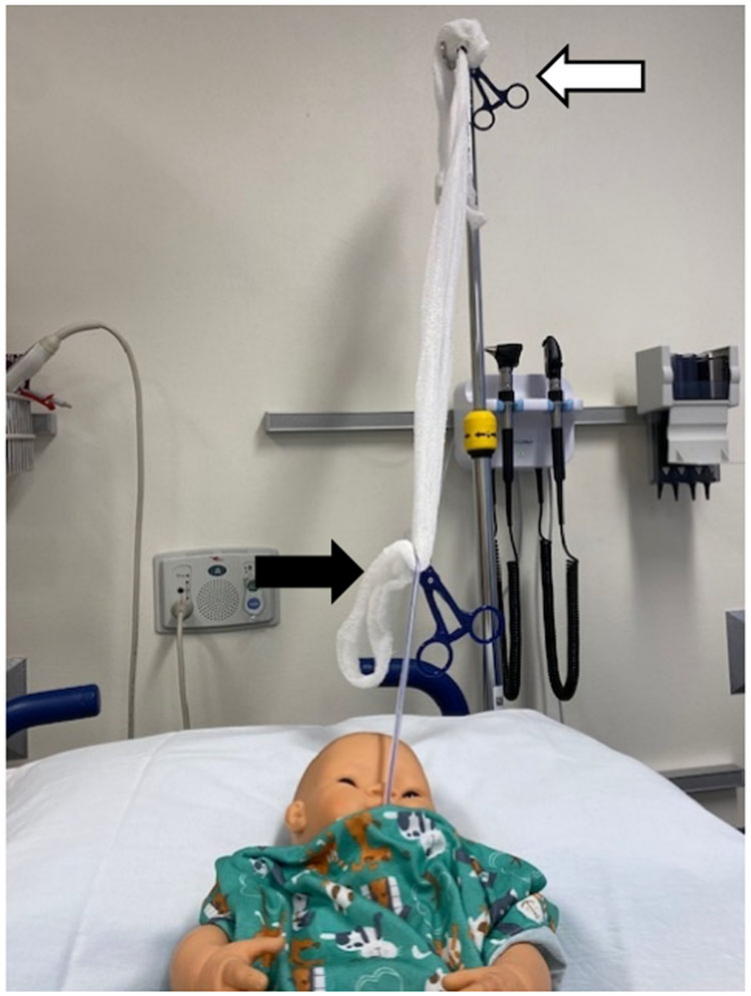
Model illustrating patient with a 12 French foley catheter clamped externally to Kerlix gauze (black arrow), looped around an intravenous pole and a second hemostat (white arrow) used to clamp gauze to itself to maintain tension.

**Figure 3:**
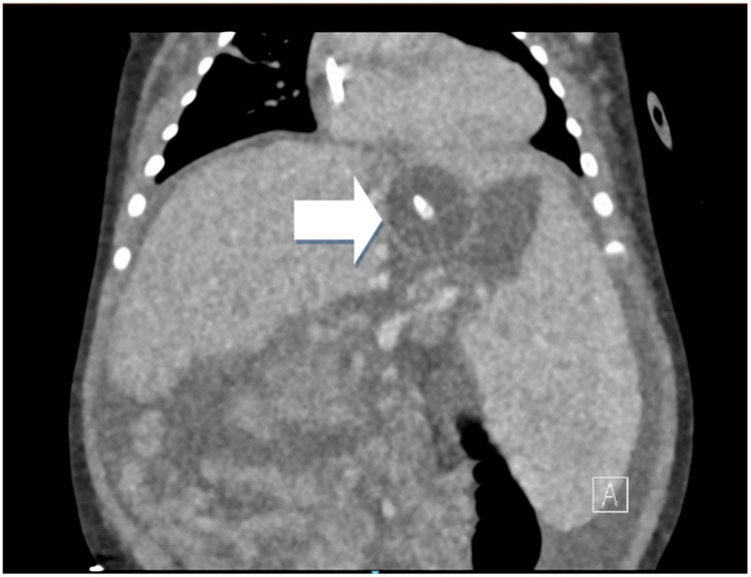
Computed tomography angiography obtained for evaluation of splenic renal shunt with visualization of foley catheter balloon inflated at the gastroesophageal junction as demonstrated by the arrow.

The patient remained stable and without evidence of re‐bleeding for approximately 48 h but then again developed bleeding from his g‐tube, associated with tachycardia and hypotension. He was provided an octreotide bolus of 2 mcg/kg, and his infusion rate was increased to 2 mcg/kg/h. Bedside upper endoscopy was performed in the ICU and was again notable for four esophageal varices, improved from prior (only 1 grade 3 varix), without evidence of active bleed (Figure 1B). A moderate amount of bright red blood was identified in the stomach and was cleared by irrigation and intermittent suction via g‐tube. A large clot was encountered at the gastroesophageal junction/gastric fundus, consistent with location of the previously identified gastric varix. Attempts were made to dislodge the clot, and evidence of clot reformation was noted. Given the patient's age and size, endoscopic interventions for bleeding control remained limited. Therefore, the decision was made to place a 12 French foley catheter in an orogastric manner for balloon tamponade, with the goal of creating a size appropriate mimic of a Minnesota tube. Location of foley catheter was confirmed via endoscopic visualization before inflation of the foley balloon with 7 mL of water, the volume of which was determined before insertion to ensure symmetric inflation of the balloon to a subjectively appropriate size. Utilizing a hemostat, the external end of the foley catheter was clamped to Kerlix gauze. The opposite end of the Kerlix gauze was then looped around the hook of an IV pole, and a second hemostat was used to clamp the gauze to itself to maintain subjectively appropriate tension and, ultimately, pressure against the bleeding varix (Figure 2). This method for balloon tamponade was successful in controlling the patient's acute bleed and placement was confirmed in a subsequent computed tomographyangiography which was performed for evaluation of splenic renal shunt (Figure 3).

### 2.3 Transfer

This intervention was recognized to be a temporizing measure until patient transfer to a transplant center was coordinated. Given the risk of pressure necrosis of the gastric tissue, the foley catheter was released from tension for 1 min every 4–6 h. After approximately 24 h, during which the patient was without evidence of re‐bleeding, the foley catheter was removed from tension but left in place with the balloon inflated in case acute rebleeding was noted during transfer. The patient remained stable without additional bleeding episodes, and he was ultimately transferred to a pediatric liver transplant center for higher level of care. We hypothesize that the use of the foley catheter in this novel manner to provide balloon tamponade at the gastroesophageal junction in conjunction with the increased octreotide dose, provided stability to reduce the risk of re‐bleeding. The patient ultimately received an unrelated living donor liver transplant 4 months later without further episodes of bleeding.

## 3 DISCUSSION

Gastroesophageal variceal bleeding, the most severe complication of portal hypertension, requires prompt intervention to prevent exsanguination and death. The preferred modality for treatment of bleeding esophageal varices includes ligation, however, this technique is limited by patient and endoscope size such as in this case. Intravariceal sclerotherapy may be used with agents such as Etanolamine oleate or Polidocanol. Associated side effects include ulcerations, as well as stricture formation.^5^, ^6^ Tissue adhesives such as N‐butyl‐cyanoacrylate may be used for bleeding gastric varices, though in this case were not available. Due to the concern for patient decompensation, the decision was made to proceed with hemostasis via a tamponade approach. Tamponade approach involves the use of balloon tubes for temporization while more permanent solution is sought to achieve bleeding hemostasis. A 12 French Blakemore esophageal balloon tube (Becton, Dickinson and Company) exists, which may have helped, but it was not available at our center. Given this, the decision was made to tamponade with the use of a Foley catheter placed in an orogastric manner and to tension for balloon tamponade, that served, in this instance, as a valid and life‐saving option for stabilization of a small infant with actively bleeding gastric varices. This intervention, along with medical management with the increase in octreotide dose, allowed for successful bleeding control and ultimately provided the time necessary to transfer the patient for further evaluation and treatment of his underlying disease process. It should be noted that with any tamponade approach, risks include pressure necrosis and perforation of the underlying tissue. Due to this risk, the tension of the inflated foley balloon against the gastric tissue was released every 4–6 h, similar to the concept used with a Minnesota tube. This methodology of catheter balloon tamponade to address active variceal bleeding in a very small infant despite other standard interventions (like octreotide) should be used only as a temporizing measure and may be considered in similar cases where evidence‐based interventions are limited.

## CONFLICT OF INTEREST STATEMENT

The authors declare no conflict of interest.

## ETHICS STATEMENT

Written informed consent was obtained from the patient's mother.
